# The role of culturally responsive social and emotional learning in supporting refugee inclusion and belonging: A thematic analysis of service provider perspectives

**DOI:** 10.1371/journal.pone.0256743

**Published:** 2021-08-26

**Authors:** Cyril Bennouna, Hannah Brumbaum, Molly M. McLay, Carine Allaf, Michael Wessells, Lindsay Stark

**Affiliations:** 1 Department of Political Science, Brown University, Providence, Rhode Island, United States of America; 2 Brown School, Washington University in St. Louis, Saint Louis, Missouri, United States of America; 3 Qatar Foundation International, Washington, Columbia, United States of America; 4 Mailman School of Public Health, Columbia University, New York, New York, United States of America; University of Copenhagen: Kobenhavns Universitet, DENMARK

## Abstract

Young refugees resettled to the U.S. from the Middle East and North Africa (MENA) region face significant acculturative stressors, including language barriers, unfamiliar norms and practices, new institutional environments, and discrimination. While schools may ease newcomer adjustment and inclusion, they also risk exacerbating acculturative stress and social exclusion. This study seeks to understand the opportunities and challenges that schoolwide social and emotional learning (SEL) efforts may present for supporting refugee incorporation, belonging, and wellbeing. We completed semi-structured interviews with a purposive sample of 40 educators and other service providers in Austin, Texas, Harrisonburg, Virginia, and Detroit Metropolitan Area, Michigan as part of the SALaMA project. We conducted a thematic analysis with transcripts from these interviews guided by the framework of culturally responsive pedagogy. The findings revealed that students and providers struggled with acculturative stressors and structural barriers to meaningful engagement. Schoolwide SEL also provided several mechanisms through which schools could facilitate newcomer adjustment and belonging, which included promoting adult SEL competencies that center equity and inclusion, cultivating more meaningfully inclusive school climates, and engaging families through school liaisons from the newcomer community. We discuss the implications of these findings for systemwide efforts to deliver culturally responsive SEL, emphasize the importance of distinguishing between cultural and structural sources of inequality, and consider how these lessons extend across sectors and disciplinary traditions.

## Introduction

Despite repeated federal efforts to restrict refugee resettlement since 2016, the United States (U.S.) received some 30,000 refugees in 2019, about a quarter of all resettled refugees around the world [1, 2]. Given a national climate with increasingly common expressions of anti-immigrant sentiment, especially towards newcomers from the Middle East and North Africa (MENA) region, and reduced federal resources for resettlement during the Trump administration, practitioners and scholars in the U.S. have struggled to support resettled refugees adequately [3, 4].

Newcomers may face significant challenges acclimating to a new society, including language barriers, unfamiliar social norms, values, belief systems, practices, institutional environments, and unwelcoming contexts of reception [3, 5]. Acculturation describes the multidimensional process of cultural exchange and transformation that takes place when newcomers and receiving societies interact [6]. Over the course of migration and resettlement, newcomers may embrace and draw strength from a plurality of cultural identities, whether on the basis of place, race, ethnicity, religion, nation, or otherwise. However, managing various cultural affiliations may also produce stress, especially in an unsupportive environment, provoking feelings of anxiety, isolation, depression, and other effects on mental health and psychosocial wellbeing [4, 7, 8]. Acculturative stress may be exacerbated by the receiving community, through microaggressions or discrimination, and by the heritage community, when some members disapprove of an individual’s process of change [9]. In the U.S., newcomers from the MENA region, in particular, may encounter identity-based discrimination, such as Islamophobia and anti-Arab sentiment, while also being subject to racialization [10, 11]. Additionally, refugees with prior exposure to armed conflict and forced migration may continue to suffer from prior adverse life events, such as witnessing violence, family separation, and protracted displacement [12, 13].

Acculturation processes may vary between individuals and across the life course. Adolescent acculturation is important to consider given the unique protective, promotive, and risk factors associated with this phase of development [10]. Some newcomer adolescents adjust to their new environments relatively quickly compared to adults, due in part to proximal supports from public services, like schools, and peer networks [8]. Adjustment may also vary depending on age at arrival, with those arriving younger tending to have higher high school graduation and college matriculation rates that are comparable to U.S.-born students [14]. The differences in acculturation between adolescents and their adult caregivers may produce a “familial acculturative gap,” during which parents remain more connected to their heritage culture, while children adapt more readily to their new society, at times distancing themselves from their heritage culture [8]. In addition to managing these acculturative stressors, resettled refugees have often had interruptions in schooling, which, when coupled with mental health and psychosocial stressors associated with displacement and resettlement, may adversely affect academic performance, belonging, and risk of dropout [8].

While common definitions of acculturation acknowledge the roles that both newcomer and receiving communities play in the process [5], research has typically focused more on the experiences and actions of newcomers [15]. However, the context of reception, including public service systems in the receiving society, such as resettlement offices, healthcare centers, and schools, are also central to acculturation [9, 15]. In addition to meeting basic needs, public service providers can also adjust their practices to promote inclusion and belonging in a process that has been termed “bureaucratic incorporation,” introducing newcomers to social networks and institutional norms and bolstering the protective and promotive factors that make adolescence a sensitive period for building resilience [16–19]. At the same time, however, public services may also produce acculturative stressors and exacerbate inequalities for newcomers, especially when service delivery systems are designed around a dominant ethnic group—such as the white, English-speaking, Judeo-Christian majority in the U.S.—or a monolithic concept of a static minority group. Such services may heighten social exclusion, contribute to marginalization, or reinforce a pressure to assimilate to the receiving society’s dominant group [8, 20, 21].

Discrimination towards immigrants and historically racialized minorities—whether in medicine, psychology, or education—has driven a growing yet fragmented research base centering the need for more welcoming and inclusive public services [22–24]. Despite these efforts, there is no cross-disciplinary, unified framework for developing or analyzing culturally responsive public services. Further complicating efforts to design and study culturally responsive service provision are close relationships between intersecting types of inequality, whether based on ethnic identity, socioeconomic status, or legal status, which are often conflated by service providers and scholars alike [25, 26].

In order to overcome such conceptual and analytic challenges, the education field has advanced a framework of culturally responsive pedagogy (CRP) [27, 28]. Originating as a response to systemic racism in U.S. education—where increasingly diverse student bodies are taught by predominantly white, female educators—CRP is not a single adaptation or intervention, but an approach that reimagines the entire educational system [29]. The framework builds on decades of education scholarship aimed at engaging historically excluded learners in the U.S., especially Black youth, by promoting equitable academic success, positive social identity formation, and an ability to grapple with social inequalities [30]. CRP proposes a systemwide approach recognizing the strengths of students from all backgrounds, drawing on cultural assets, life experiences, and learning styles to promote more inclusive education [31] and demanding change of faculty, staff, curricula, policies, and processes for this effort [29]. Increasingly, educators are calling for practices that not only maintain students’ cultural heritages—as if those were static—but also sustain and respect them through continued engagement, such as through native language instruction [32].

CRP offers a useful framework for supporting newcomer acculturation through systems and practices that attend to social identity, belonging, and equity. While research on culturally responsive services has rarely intersected with the acculturation literature, this relationship is emerging, as culturally responsive programs are increasingly used to help mitigate acculturative stress [33–35]. In this article, we draw on CRP to study how educators and other school-related service providers participate in acculturation with adolescent newcomers from the MENA region. In particular, we apply CRP to analyze schoolwide social and emotional learning (SEL), an increasingly common educational model that strives to build the knowledge, attitudes, and skills that students need for success at school and beyond [36, 37]. Although there is considerable variation across SEL approaches [38], we engage principally with the Collaborative for Academic, Social, and Emotional Learning (CASEL)’s widely used model, which centers on five “core competencies,” namely self-awareness, self-management, social awareness, relationship skills, and responsible decision-making. CASEL conceptualizes SEL at multiple levels, including core competencies for not only individual students, but also adult service providers, school climate, and family and community partnerships [39].

With this focus on cultivating awareness and healthy relationships across these levels, a growing interdisciplinary literature has recognized SEL as an opportunity to support acculturation among students and providers, while also promoting newcomer wellbeing [40, 41]. In a recent participatory study with adolescents resettled from the MENA region [35], for example, we found that, in addition to broadening SEL competencies to emphasize inclusion and equity, these students wanted more emphasis on culturally responsive teaching and on promoting a welcoming school climate. But how do educators and other service providers working with these students think about the opportunities and challenges of delivering culturally responsive SEL? What approaches do they consider successful for supporting refugee incorporation, belonging, and wellbeing after resettlement, and what are the limitations of these approaches? Guided by these research questions, this article identifies several promising approaches for promoting culturally responsive SEL across the school system, but it also investigates common barriers to inclusion that result in part from an insufficient understanding of the needs and preferences of the resettled MENA population and from inattention to the distinct cultural and structural challenges that they face.

## Methods

### Setting

Data collection took place as part of the Study of Adolescent Lives after Migration to America (SALaMA), a multi-year, mixed-methods study exploring the mental health and psychosocial wellbeing of newcomers resettled to the U.S. from the MENA region. We conducted interviews in Harrisonburg, Virginia and Austin, Texas during summer 2018 and in the Detroit Metropolitan Area (DMA) during fall 2019 [40, 42]. We selected these sites purposively, based on their histories of welcoming families from conflict-affected MENA countries and existing relationships with local school systems. Collectively, Virginia, Texas, and Michigan have resettled 123,288 refugees since 2008 [1]. In 2015, 57.8% of refugees and special immigrant visa (SIV) holders in Michigan were from the MENA region, compared to 29.5% in Virginia, 20.2% in Texas, and 15.6% nationally [43].

Harrisonburg City Public Schools (HCPS) serves around 6,400 students, 46% of whom were born outside the U.S. Around 9% of students speak Arabic, and 6% speak Kurdish [44]. Austin Independent School District (AISD) serves around 80,000 students, 27% of whom are English language learners (ELLs), with Arabic being the most common language after Spanish [45]. DMA is well-known as having among the largest Arab ethnic enclaves in the U.S. [46]. Global Educational Excellence (GEE), a network of charter schools, serves approximately 4,500 students across 14 schools in Michigan and Ohio, as well as seven schools in the Middle East [47]. At the three GEE high schools in this study, about 53% of students are ELLs, with a significant percentage being newcomers from conflict-affected countries in the MENA region [48].

### Participants

This article focuses on the subset of SALaMA data involving key informants responsible for services, programming, or policies relevant to newcomer adolescents and their families. Key informants included teachers, guidance counselors, school district/division administrators, case workers, therapists, and NGO personnel, which we refer to broadly as “service providers.” Several also offered newcomers a range of psychosocial supports that extended well beyond their formal responsibilities [40]. In all three sites, we selected key informants purposively from lists of service providers developed with local partners (in Harrisonburg, a division administrator; in Austin, AISD’s Refugee Family Support Office; in DMA, a GEE system official and support staff). The research team recruited from the lists via email and/or phone and continued recruitment until reaching saturation, which we assess as being the point where little new relevant information being shared. All service providers recruited for this study agreed to participate or referred us to a colleague who was deemed to have more relevant information.

We developed separate semi-structured interview guides for use with educators, other service providers, administrators, and government officials, respectively (S1 File). Guided by an overarching aim of understanding how schools and their community partners support newcomers from the MENA region, the study team designed these semi-structured interview guides by reviewing available public information about the research sites (e.g., school and resettlement office websites, local newspapers, and published reports), conducting pre-visit consultations with local partners, and conducting preliminary field visits. In all sites, key informants provided written informed consent and were asked to recommend other potential participants.

### Data collection

Data collection consisted of semi-structured interviews lasting 30–90 minutes. Interviews typically took place in the participant’s office or in a similar professional setting where privacy could be secured. The data collection team consisted of four public health researchers, with two conducting interviews in Harrisonburg and Austin and two conducting interviews in DMA. All researchers were trained qualitative interviewers and had received special training on best ethical practices for research on forced migration. Questions were tailored to key informants’ job functions and focused on challenges faced by newcomers, as well as available supports, strengths that newcomers brought to their schools and communities, and ideas for promoting positive outcomes for these students. The researchers recorded all data collection sessions using an audio-recording device, unless the participant requested otherwise, and kept detailed field notes throughout. Following the recordings, team members transcribed all audio files. The research team then reviewed, edited, and de-identified all transcripts, which were only available to the analysis team.

### Ethical considerations

The Austin and Harrisonburg research protocols were approved by the Institutional Review Boards (IRB) at Columbia University’s Mailman School of Public Health (IRB-AAAR7830), AISD’s Department of Research and Evaluation (R18.62), and the Superintendent of Schools at HCPS. The DMA protocol was approved by the Institutional Review Board (IRB) at Washington University in St. Louis (IRB-201905151) and participating schools’ principals.

### Data analysis

Taking a grounded theory approach, we conducted thematic analysis with transcribed data using the constant comparative method [49, 50]. After reviewing transcripts, the research team developed initial codes corresponding to research objectives and questions. We then refined these codes using existing literature on immigration acculturation and incorporation, social and emotional learning, and mental health and psychosocial wellbeing to develop analytic memos and an initial codebook that grouped the codes into larger themes corresponding to the literature (i.e., life experiences and challenges; school-specific; supports; relationships; structural factors; intercultural factors; linkages; innovations). We then recruited a team of six researchers with backgrounds in public health, social work, psychology, and refugee resettlement to code data after being trained on the study protocol and codebook and they further refined the codes and themes. After iterating the codebook through multiple rounds of review and revision, the team finalized the codebook’s 62 codes and eight themes and built inter-coder reliability (ICR) using Dedoose’s Training Center, during which each member of the team double blind-coded segments of the dataset. Coders were required to reach at least 66.7% ICR on tested codes before being assigned transcripts in Dedoose. Coding applications were reviewed by the lead analyst to resolve questions and issues.

To better understand the ways in which service providers delivered culturally responsive interventions to support acculturation, team members utilized another iterative process to identify themes and sub-themes in the data related to adult SEL, school climate, and family partnerships. They compared these to empirical and theoretical literatures on adolescent SEL, its various adaptations, and the mental health and psychosocial wellbeing of forced migrants.

## Results

Forty service providers were interviewed across the three sites (Table 1). Of the providers, 65% (n = 26) identified as women; 55% (n = 22) were in leadership positions (e.g., administration), while 45% (n = 18) were in direct service provision roles. School-based providers comprised the majority of the sample at the district (n = 9) and school (n = 16) levels, with 37.5% of the sample consisting of community-based providers (n = 15). No participant dropped out of the study and one participant was interviewed twice.

**Table 1:** Key informants by location, position, and gender.

	Austin	Harrisonburg	Detroit Metro Area	Total
Total # of Providers	17	10	13	40
Male	5	4	5	14
Female	12	6	8	26
Affiliation				
School	5	8	3	16
District	4	5	N/A	9
Community-based organization	8	5	2	15
Position				
Service provider	5	3	10	18
Leadership	12	7	3	22

*Note*. District level was not an applicable category in DMA.

**Fig 1:**
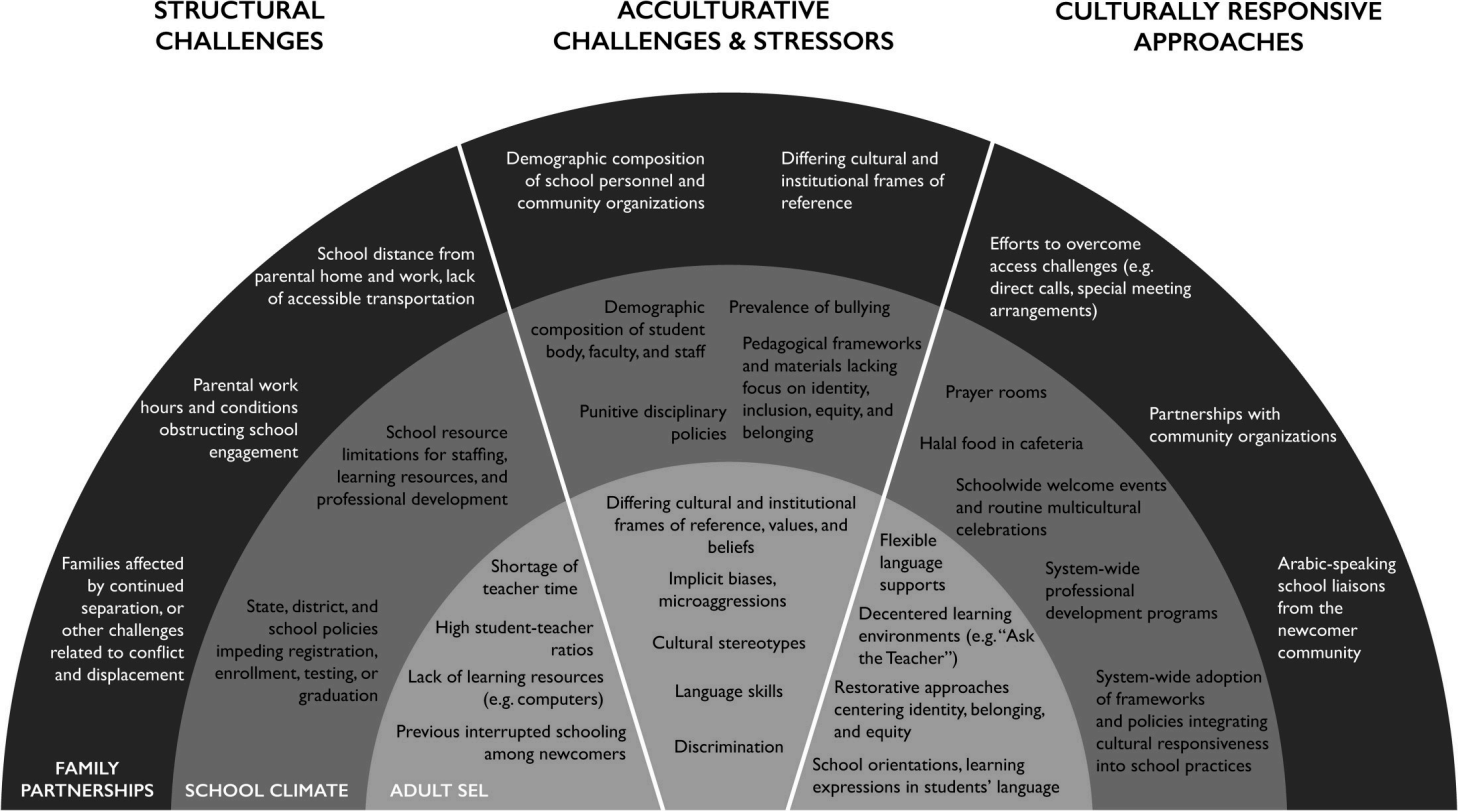
A framework for understanding culturally responsive SEL in the context of refugee resettlement.

Service providers articulated challenges experienced in providing culturally responsive services that supported newcomer students’ unique needs during acculturation. These challenges arose 1) in the social and emotional learning of service providers (adult SEL); 2) in the design and implementation of policies and initiatives (school climate); and 3) in building partnerships with newcomer families (family partnerships). Service providers also identified various forms of culturally responsive efforts that they took to address these challenges and support newcomer students at each level (Fig 1).

### Adult social and emotional learning

Service providers discussed several challenges and successes in providing culturally responsive supports for newcomers. Some providers struggled to understand and prioritize newcomer students’ underlying needs, often focusing instead on immediate challenges, such as language barriers. Many, however, moved beyond identifying these proximate academic barriers, reflecting on the underlying acculturative challenges that hindered school participation and sense of belonging. As one district leader in Michigan offered,

[K]ids come from different environments… bring different baggage with them to school, and if the school culture, the school system, and the school environment is not trained enough to unpack, to help this child open their bag… I don’t think that we are meeting the needs of the students. (KII_M1.13)

Several participants were also concerned about newcomers having experienced prior trauma, described by one Harrisonburg provider as “specific social emotional needs… derived from coming from a war-torn country or being a female in a certain refugee camp” (KII_H1.04). In recounting these perceived needs, providers occasionally perpetuated stereotypes associated with refugees, Arabs, or Muslims, with varying degrees of self-awareness of their own biases. For instance, one Michigan provider explained that newcomer boys sometimes resisted personalized teacher support because of their so-called “Arabic mindset,” elaborating that, because of “their ego, they can’t be special or singled out” (KII_M1.04). Others, such as this Austin provider, noted a perceived conflict between what they considered to be different newcomer groups, alarmed about “families being placed together who have hundreds of years of historical conflict, and then putting them in the same classroom… to start learning right away” (KII_A1.11/12).

Other providers were more adept at recognizing, even challenging their biases, such as one Harrisonburg district leader who cautioned, “Just look at each [student] as unique. Do not mainframe them or do not stereotype them” (KII_H1.04). For this participant, culturally responsive education meant addressing each student as an individual, rather than as a representative of a general group. Sometimes, such insights led providers to reflect on their own privilege. One Michigan teacher shared:

…I was from my very white-centered perspective, and thinking, “Oh, I’m learning Arabic and all these things.” And having that humble moment like, “Oh, maybe I’m not being as clear with my teaching. Maybe I’m not really teaching as well as I thought I was” …. My kids who know me, my phrase is: “Is this some crazy white people stuff?” Because sometimes I’ll give them a reference, and I’ll check myself, because as it’s coming out of my mouth, I’m like, “No. They have no frame of reference.” (KII_M1.10)

This participant recognized where gaps in understanding may have been fueled by contrasting cultural frames of reference and adjusted their teaching as a result.

Providers demonstrated various SEL-informed ways in which they met challenges, overcame biases, and provided services to support newcomers. Regarding shorter-term strategies, faculty in several schools offered walkthroughs to orient newcomers to their new school environment, explaining features like fire alarms and appropriate school attire. Educators also modeled welcoming practices for their students. In Austin, for instance, a participant recalled how a teacher had not only emphasized the importance of pronouncing an incoming student’s name correctly but had also brought in learning materials from the student’s country of origin, teaching the American students to say “welcome” in Farsi. In the participant’s opinion, “that teacher was really making sure that child was going to be embraced in that educational setting” (KII_A1.08).

Providers observed that many newcomers “have the academic chops… the motivation… the work ethic… [to] be succeeding like crazy in these courses,” in the words of one Harrisonburg participant, but often lacked the appropriate language support for meaningful engagement (KII_H1.08). Several educators expanded language support options to be more culturally responsive, such as by providing diaries where students could write in multiple languages, making test adjustments, employing visual aids and games, and offering one-on-one attention during group work. A few providers took these practices further by challenging didactic instructional norms and leveling classroom power dynamics, such as one Michigan provider who hosted “Ask the Teacher” moments, where students could pose questions and begin building relationships in a more egalitarian environment (KII_M1.09).

In several schools, providers used restorative practices centered on nurturing healthy relationships, creating just and equitable learning environments, and repairing harm due to conflict or perceived misbehavior. Teachers often described their shift away from policing toward “peacekeeper” (KII_M1.12), offering students space to process and share their experiences. These approaches countered punitive practices for behaviors potentially resulting from acculturative stress and prompted providers to reflect on their own cultural frames, at times leading to greater understanding of their students. In one such instance, a Michigan teacher realized why a male student felt uncomfortable making eye contact with her and changed her approach to make him more comfortable. She shared:

If I have a kid who’s reacting to something because this is what he has been socially inculcated with, there is no way that I’m gonna overcome that… by putting him in detention… I spend the first two weeks of my school year developing relationships because I want the kids to know that this is a safe place… (KII_M1.09)

Providers also used culturally responsive restorative practices with students who mistreated newcomers, such as a boy in Austin who pulled off a student’s hijab (KII_A1.13). The teacher engaged in harm reparation by asking questions and encouraging honest dialogue between the students, halting future incidents and leading to increased confidence and wellbeing in the student who was harmed. The teacher recalled asking the offending student, “why did you do it?” and the boy responding, “I just didn’t… I was wrong, I shouldn’t have done it.” The teacher went on to explain:

[H]e was trying to see what’s underneath [the hijab]… since we [were] able to shut it down real fast, that little girl, now she runs up and down these hallways… she smiles, ‘Good morning!… when it comes to cultural pieces like that, that’s how we approach… because I’m a big advocate of restorative practice—it’s understanding the “whys” behind it. (KII_A1.13)

Many providers demonstrated their commitment to cultural responsiveness by centering newcomer students as experts in their education. Some providers, for example, created listening spaces focused on students’ priorities, an exercise a few educators referred as “co-creating.” Others went further, letting students take the lead. As one Michigan teacher shared,

…it’s made me think about teaching in a different way. I was never “sage on the stage” … But, working with this particular group of students, I’ve really learned how to put the teaching back on them… it’s not so much about me being the disseminator of knowledge, but me being the resource in the room that helps them learn to learn. (KII_M1.10)

### School climate

Service providers reported a variety of schoolwide measures to ensure that programs and policies were culturally responsive to newcomers. Participants across sites also reported common resources constraints that hampered these efforts to varying degrees, including unmet translation needs, high student-teacher ratios, understaffed paraprofessional support, and a lack of learning materials like computers. In some cases, broad school, district, and state education policies also presented challenges for newcomers, whether in registering for classes, completing standardized exams, or meeting graduation requirements.

Schoolwide responses often started with special programs, such as multicultural celebrations and welcome events. Newcomer-focused initiatives in some schools, however, led to larger-scale systemic changes. One Austin school was “really intentional” about having a “halal line” in the cafeteria (KII_A1.06). A Harrisonburg school converted a conference room into a designated prayer space during Ramadan, created and advertised by the students and facilitated by local mosque leaders. These initiatives elevated schoolwide efforts from static concepts of cultural competence to active processes of cultural exchange.

Schoolwide SEL initiatives usually included efforts both to support educators’ own SEL through professional development and to integrate SEL for students throughout classrooms and programs. Several providers expressed a particular need for greater schoolwide and even systemwide investments in culturally responsive adult SEL. In one Austin administrator’s words:

…adult SEL really needs to come first… We can’t ask our teachers to really model and teach socioemotional strategies to students if they can’t manage their own emotions, or don’t understand their own identity or can’t build relationships themselves… It goes all the way up… from the boardroom to the living room… We need to make sure we’re extending this growth effort at all levels of leadership in the district…. because if you haven’t examined your own beliefs around immigrants, refugees, Muslims, people of color… people who speak Arabic… there’s gonna be some question about how you’re serving them in your school. (KII_A1.01)

A Michigan provider relayed a similar message about schoolwide cultural responsiveness, explaining, “I’m not talking about being an expert in all the cultures of your students that you are teaching… actually, you cannot be…. [We] should establish the culture in the school that we want to learn about each other” (KII_M1.13).

While a few providers thought that schoolwide SEL programs would provide “something different” for newcomer students in particular (KII_M1.01), others emphasized the need to “talk about [SEL] as whole community issues, and not, like, ‘these kids are special or broken’” (KII_H1.07), in the words of one Harrisonburg participant. For these latter providers, SEL offered an opportunity for the entire school to create a climate of safety and inclusive support. A Michigan teacher articulated the schoolwide SEL message they aimed to convey: “My teacher is here for me, mentally and emotionally first. Where I’m at a 100% safe zone. And then he’s here for me, she’s here for me, to educate me” (KII_M1.04).

Trainings were one of the most common schoolwide SEL initiatives, and they varied in perceived scope and success in each site. Several providers thought of SEL trainings as opportunities to discuss ways to support newcomer students. Others found that SEL implementation was too superficial, that trainings were one-off and unmemorable. Where routine trainings were available, providers sometimes felt overwhelmed and stretched thin, with too many competing priorities to be able to invest in ongoing professional development. In Austin, SEL trainings were integrated in a new systemwide model focused on equity, power, identity, and belonging. As one Austin district leader explained, compared to conventional SEL, this “SEL 2.0” included:

…a more explicit focus on equity around race and… economics… a more explicit understanding that there’s SEL, and [an] upper middle class, white suburban neighborhood can look very different from what SEL is in a neighborhood that has lots of refugee students or that’s predominantly African-American or that has lots of poverty… Understanding your own identity linguistically and culturally… having the cross-cultural, cultural competence to make the connections and relationships with others who are different from you. (KII_A1.01)

Several schools had begun employing frameworks around restorative justice and trauma-informed care as well, though there were fewer formalized programs compared to schoolwide SEL. Providers mentioned policies that centered healthy relationships through practices such as primary prevention, conflict de-escalation, and mediation, rather than conventional zero tolerance policies that favor punishment. For instance, students in one school had “a space where kids can go… instead of sending them to the office, if they are angry or they shouldn’t be in the classroom because they are misbehaving, then they could go to this room” to relax (KII_M1.06). Multi-level behavioral support strategies were in place at some schools, connecting teachers, administration, and social workers in these efforts.

On occasion, restorative justice was embedded into the school’s operations at all levels—from classroom training, to specific policies, to considerations for student-school communication—such as this Michigan school’s preventative response to bullying:

…we push into every single classroom and we tailor it to their age, to their grade level, and we go over what bullying is, what an upstander/bystander is. Various methods to communicate to your teachers or office staff and administrators. We ensure them that we’re here to support you…. I tell the students that if you don’t want to talk to a teacher… you write a note and stick it underneath the door. Or… they email me or whoever they feel comfortable emailing, and we deal with the bully…. and if we notice that the bully needs counseling, then we sit with the parents and start designing that plan. (KII_M1.04)

In this instance, schools gave students the flexibility to report to school personnel via mechanisms that felt most comfortable to them, reducing barriers to reporting, strengthening relationships with providers, and centering student autonomy, while also prioritizing mental health and psychosocial support for the student using harm, rather than punishment. Such efforts also strengthened SEL competencies, while considering their needs and behavior in the context of their previous experiences, helping providers to “understand kids and why they might be acting a certain way” (KII_H1.04).

### Family and community partnerships

A crucial support for newcomer students involved school engagement with families and communities, which helped to narrow the acculturative gap between adolescents and their adult family members. One Michigan school leader viewed the school as a “community learning center,” stressing the importance of “the students feel[ing] there is a bond between the family and the school” (KII_M1.03). While providers identified several challenges that newcomer families faced when engaging school systems, they also identified ways in which the school and community partners successfully partnered for the benefit of students’ educational growth and sense of belonging.

Common challenges that newcomer families encountered included access to stable jobs and decent incomes, affordable and safe housing, and transportation. However, providers also discussed a number of intercultural challenges in family engagement. In addition to language barriers, providers often felt that “it’s a challenge to get parents to understand what the system is and how to make the system work for them,” in the words of one Harrisonburg participant (KII_H1.04). Such challenges may have resulted in part from institutional differences between national education systems and a range of differences related to school norms (e.g., dress code), practices (e.g., homeroom), values (e.g., SEL), and parental roles in education. Providers noted that, while parents “see us as the experts… they’re the parents” (KII_M1.09) and there is a limit to what schools can accomplish without their partnership—a perspective on parental roles in education that may have been new to some parents.

Providers also struggled to navigate perceived cultural differences in their interactions with families, such as with gender norms and roles, which vary within and across MENA groups. These providers noted that women caregivers sometimes could not attend events alone, meet with a male teacher or administrator, or drive, which limited school engagement. Providers sometimes had difficulty explaining restorative and trauma-informed practices to families, as articulated by this Michigan provider:

It’s just, some of these kids, when they realize that school here is not gonna hit them, they act out a lot more… And we have a hard time getting the families to understand that, ‘No, we are not gonna hit your child… but, they are still expected to do x.’ And the parents don’t understand, ‘Well, if you’re not gonna hit them, how are you gonna get them to do it?’ (KII_M1.10)

Engaging families in students’ SEL often involved explaining the “why” behind the model to families in a culturally responsive way. In the words of one Austin provider, the challenge was “to introduce [SEL] and talk about it and not make it a taboo” (KII_A1.04).

Providers respected families’ commitment to sustaining newcomer students’ connection with their heritage cultures, while also depending upon families to help minimize acculturative stress. They reported difficulties in striking this balance. The message for parents, according to one Michigan provider was:

…we want you to teach Arabic to your children… that’s hugely important to your culture and your family. But we also need you to understand that, as long as they’re here in the United States, they’re gonna function better if they understand English (KII_M1.09).

Despite these challenges, providers utilized several strategies for engaging families meaningfully. A necessary, if insufficient, first step, according to several participants, was to recognize the obstacles and devise intentional supports to make schools more accessible for newcomer families. In the words of one Harrisonburg district leader:

…when we have such a diverse community—over 70 languages—cultural background and understanding of the responsibilities and roles of a parent or a family within a child’s education is very different. So, we really try to find a way to make it easy to navigate, easy to understand for families, simple ways to get involved, and really becoming very intentional in looking at… some of the barriers that typically have prevented families from coming into our schools, and how to remove those and create a more accessible means of interacting with us… (KII_H1.06)

One innovative way that several schools mitigated these challenges was by hiring paraprofessional liaisons that shared cultural heritage with newcomer students. According to one Harrisonburg participant, these liaisons served a variety of functions: “they interpret; they translate; they advocate for families; they serve as a bridge between the community and the school” (KII_H1.06). Across study sites, Arabic-speaking liaisons from the MENA region, some of whom had also been resettled as refugees, frequently met with families and fostered connections with community organizations, aiding newcomers with services ranging from tutoring, language courses, and daycare, to mental health and psychosocial support. An Austin district leader described the role of the school liaison as “helping the families in a way that is respectful to the system we’re working in. Relationship-building. Collaboration. Listening. Making mistakes and learning from them” (KII_A1.03).

Liaisons were not the only bridge offered to families. In many schools, family members were connected to individual teachers, social workers, and administrators. In Michigan, for example, participants explained that one of the principals had developed strong ties with the community. In one teacher’s words:

I have seen these parents… keep in contact with the principal… He was an immigrant from before, and he kinda gets them and he gets where they come from… He works with them and he does his best to make it comfortable for them… it’s just, it’s very like a close community… (KII_M1.01)

While this participant was surprised to see families with the principal’s phone number, they noted that this close tie between families and administrators led to increased belonging.

Through these school-facilitated connections, in the words of one Austin provider, “…to have the school initiate those conversations and say ‘we see you and we see your child and we have resources for you’… then it really roots in the community, and [families] feel empowered…” (KII_A1.12/11). Providers benefited from these connections also, as they came to understand and honor the vast contribution that families have to offer. In the words of one Harrisonburg district leader:

We recognize that they have knowledge and wisdom and they have all these assets that they need to be part of a conversation, right? And they not only need to be invited, but they need to be included and they need to feel like they have a share, you know, responsibility at the table, and that their input is greatly appreciated. (KII_H1.06)

These partnerships centered the cultural exchange between providers and families as they worked together to support newcomer students.

## Discussion

This study illuminates the central but understudied role that public services—specifically schools—play in refugee incorporation and acculturation after resettlement. Across study sites, schools and providers attempted to support newcomer adjustment while both adapting to and learning from their new students. The SEL initiatives being implemented in these schools, with their focus on promoting strong relationships founded on social awareness and empathy, provided a unique opportunity to observe how educational practices at the level of individual provider (adult SEL), school (school climate), and community (family partnerships) responded to the needs and preferences of newcomers from the MENA region. Guided by the framework of culturally responsive pedagogy, we identified several promising approaches that may merit further examination, but also systemwide shortcomings in school responsiveness to these students and their families. Below, we discuss implications of these findings for efforts to deliver culturally responsive SEL, draw attention to the critical importance of distinguishing between cultural and structural sources of inequality in devising and delivering responsive supports, and consider how these lessons extend across sectors and disciplinary traditions.

### Going all the way up: Cultural responsiveness from the student to the system

Considerable variation in school and provider supports for newcomers points to the importance of integrating cultural responsiveness into systemwide SEL efforts at the level of the district/division and perhaps beyond. CASEL, whose universal model informed SEL initiatives in several study sites, has begun to transform its approach by adopting an equity lens that centers social identity, cultural assets, the role of power, and belonging [51]. Efforts to map the various models beyond CASEL have drawn attention to additional opportunities for integrating these principles into SEL initiatives [38]. Findings from this study attest to the critical value of implementing transformative approaches that take newcomers into account, not only at the student curricular level, but also in adult SEL, in school climate, and in community partnerships.

While the burgeoning literature on bureaucratic incorporation underscores the importance of professional norms in cultivating more welcoming, inclusive services, our findings indicate that the norms underpinning cultural responsiveness had not been evenly understood or adopted within the selected schools [18–19]. The continued tendency among even the most well-intentioned service providers to misunderstand newcomers and resort to cultural stereotypes in our sites indicates a need not only for greater diffusion of welcoming policies and sustained CRP professional development, but also for routine self- and climate-assessments. Such measures would help to ensure that individuals and systems are not doing harm, but instead nourishing a sense of belonging among all students [8]. Indeed, several providers who had benefited from culturally responsive adult SEL programming reportedly questioned their own frames of reference and biases, actively learned from resettled students, and committed themselves to more flexible, inclusive pedagogies—a pattern that warrants further study.

Efforts to promote an inclusive school climate also varied in their degree of cultural responsiveness. Although many participants considered practices such as multicultural celebration days and orientation walk-throughs to be promising, some also felt that such activities would fall short without lasting efforts to cultivate a schoolwide environment of cross-cultural exchange and inclusion. These sentiments find support in the education literature, where scholars have argued that symbolic, one-off events celebrating fixed concepts of culture, rather than promoting meaningful exchange or identity development, can “mask, rather than address, serious equity concerns” [52]. Examples of longer-term, systemic changes implemented by schools included halal food cafeteria lines and prayer spaces, which not only showed respect for newcomers’ traditions and beliefs, but also helped to sustain them through integration into the everyday school climate. Further, school administrations, such as HCPS, had institutionalized their commitment to welcoming newcomers by enshrining equity as a key objective in their strategy plan and issuing a division-wide statement of inclusivity [44]. Future studies should examine how systemwide efforts like these contribute to school climate and to newcomer educational and acculturation outcomes, as well as how such efforts relate to broader patterns of refugee receptiveness beyond the education system, whether in other public service systems or in the wider sociopolitical context [17]. For example, it would be worthwhile to investigate the degree to which meaningfully inclusive public service initiatives might shield newcomers from the determinantal effects of hostile policy environments and widespread anti-immigrant sentiment [53].

Finally, although education systems often struggled to engage newcomer families as a result of complex access barriers, several schools had developed innovative solutions to involve caregivers more meaningfully in student education. In particular, some schools hired and trained Arabic-speaking paraprofessional liaisons as cultural mediators that worked closely with students and teachers and brokered enduring connections with families and community organizations. Meanwhile, energetic school and district leaders learned from these liaisons in order to improve their relationships with students and their caregivers. Such examples reflect the value of systemwide investments in developing adult SEL skills related to social identity and relationship-building and in hiring personnel that reflect the makeup of the student population [37, 51, 54].

### Cultural responsiveness is also about structural inequality

A common challenge among providers was disentangling how newcomers’ needs and preferences related to their acculturation, racial or ethnic identity, refugee status, prior experiences of conflict and displacement, and current socioeconomic position, all of which were commonly misinterpreted as perceived “cultural differences.” However, these assumptions were often inappropriate, and the fixation on cultural differences clouded the underlying needs of students, a phenomenon that scholars have repeatedly pointed out in a variety of contexts [52, 55, 56]. For instance, teachers blamed students’ academic difficulties on language barriers, rather than the lack of resources to hire paraprofessionals and provide appropriate learning materials to students. Another provider evoked stereotypes about Arab masculine egos to explain why some students did not like personalized teacher attention, rather than recognizing that many students—of all backgrounds—preferred not to be singled out by authority figures. Attributing these educational outcomes to social identity or values, rather than to the fundamental causes of social, political, and economic inequality, risks placing blame on students and the groups to which they belong, while also directing scarce educational resources to solving the wrong problem. Such tendencies attest to the potential value of school administrations such as AISD investing in professional development sessions focused on identifying and responding to the unique challenges that conflict-affected populations face as they adjust to U.S. public schools [45].

In recent years, scholars and practitioners have recognized the need to address this challenge more systemically and have proposed a variety of frameworks—such as structural competency, equity literacy, and culturally responsive pedagogy—that focus on preparing service providers to recognize how structural inequality and life experiences might condition individuals’ resources, behavior, and performance [52, 57]. Our findings reflect the critical relevance of such frameworks and demonstrate the particular utility of CRP for the case of social and emotional learning. A framework born of Black educators striving to make schools more supportive for historically excluded, non-white, and especially Black American students [30, 31], CRP offers a wealth of resources for promoting inclusion and belonging among students resettled from the MENA region. Although there are parallels and intersections between the experiences of these student populations, especially as refugees of color become racialized over time in the U.S. [11], there may also be substantial differences in the experiences, needs, and preferences of resettled refugees, which warrant close attention using a culturally responsive framework. This study contributes to a growing literature expanding CRP to include newcomer students more explicitly [32], an important area for continued research.

### Significance for public health services

These findings have implications well beyond education. Cultural responsiveness is applicable to a range of public services, including medicine, psychology, and public health. These fields have often focused on narrower efforts to tailor existing interventions to new populations through cultural adaptation or to build awareness among clinicians through cultural competence trainings. As several scholars have argued, such limited conceptions of the relationship between culture and health are not only insufficient for overcoming health disparities, but also risk reinforcing static concepts of cultural identity, reproducing cultural stereotypes (e.g., of the form “x people don’t like y”), and distracting from systems-level change [7, 58]. CRP, by contrast, offers a systemic change approach that involves providers, policymakers, and communities in efforts to foster an understanding of the structural determinants of health, recognize and bolster newcomer strengths, and deliver services that respect health beliefs, values, and practices [29]. In this way, applying the insights and lessons of CRP may inform continued efforts to produce more inclusive health practices.

Given the centrality of public services in supporting the resettlement and incorporation of newcomers, further research should aim to ascertain the degree to which frontline service providers, such as resettlement offices, human services departments, workforce development boards, and health clinics provide culturally responsive supports. Researchers would do well to work with these providers to determine which culturally responsive strategies are most feasible, effective, and appropriate, given population needs and resource availability concerns. Such research would be especially beneficial for efforts to rebuild the U.S. Refugee Admissions Program under the Biden administration [59]. In order to guide the growth of this scholarship, moreover, researchers from across disciplines should aim to develop a shared cultural responsiveness framework that bridges lessons learned from education, public health, psychology, and other disciplinary traditions focused on serving diverse publics.

Several study limitations are worth noting. The study did not systematically evaluate SEL in these schools and should not be seen as an assessment of its implementation or effectiveness. Nor did the study include policymakers beyond the school district/division level, though further research with such participants would likely produce valuable insights into how public policies, budgetary concerns, public attitudes towards immigrants, and other aspects of the sociopolitical context influence refugee receptiveness in schools [17, 19]. This analysis focused only on the perspectives of service providers, principally educators, though articles reporting on the perspectives of refugee students and families in this study have been published elsewhere [35, 40, 60]. We used purposive sampling to select study sites where school districts had a relatively high degree of support for resettled refugees. Whereas this selection strategy was designed to secure safe participation, providers from schools with less structured programming for newcomer students may have reported different experiences. Thus, these results cannot be generalized to the entire population of providers working with newcomer youth from the MENA region.

## Conclusions

Schools play a central role in welcoming young refugees after resettlement, but they also risk exacerbating acculturative stress when they make newcomers feel unwelcome or pressure them to assimilate. Newcomers and service providers across study sites encountered numerous obstacles to meaningful engagement, while often also overcoming those challenges in innovative ways that sometimes recognized and drew upon the cultural assets of newcomer students and their families. This study highlights the opportunities that schoolwide SEL efforts offer for supporting newcomer incorporation and belonging through measures to enhance culturally responsive adult social and emotional learning, to cultivate more welcoming and inclusive school climates, and to develop strong relationships with newcomer families and communities.

## Supporting information

S1 File Semi-structured interview guide–key informants. This is the semi-structured interview guide used with key informants. Specific question wording and probing questions were modified by interviewers during the course of field work in order to adjust to the local context and to the information being shared by participants.
(DOCX)
