# Supplementary material for: The role of culturally responsive social and emotional learning in supporting refugee inclusion and belonging: A thematic analysis of service provider perspectives
Source: PLoS One. 2021 Aug 26;16(8):e0256743. doi: 10.1371/journal.pone.0256743 (PMC8389371; doi:10.1371/journal.pone.0256743)
Supplement: S1 File — This is the semi-structured interview guide used with key informants. Specific question wording and probing questions were modified by interviewers during the course of field work in order to adjust to the local context and to the information being shared by participants. (DOCX) [file pone.0256743.s001.docx]

**S1 File. Semi-Structured Interview Guide – Key informants**

**Study of Adolescent Lives After Migration to America (SALaMA)**

**Columbia University and Qatar Foundation International**

This semi-structured guide includes an introductory script, guiding questions, and probes for the key informant interview with school staff and personnel, government officials, service providers, and other relevant stakeholders. Please note:

- After the introductory script and informed consent, **the interview should take under 1 hour to complete**.
- The questions below simply provide a framework for the interview but remember that the discussion may lead to other important questions not listed below.
- When working with participants, please remember to use accessible and sensitive language.
- The interview should take place in a quiet and secure place, where the participant feels comfortable and is not readily interrupted or distracted.

**Introductory Script** [if conducting interview directly after informed consent, much of the below may be skipped]:

Hello, and thank you again for joining us today. I really appreciate your taking the time to speak with us. I am from Columbia University in New York and am excited to be here with you. As we’ve already discussed, we are part of a research team working with this school district on a study of student wellbeing.

One thing we’re especially interested in is how high schools serve students that have been resettled to the U.S. as refugees from countries with Arab majorities, like Iraq, Syria, and Yemen. The idea is to learn from students, caregivers, school personnel, and other leaders in the area so that we can understand what is working well for these students and what additional kinds of support they may need.

Our conversation today should last no longer than 1 hour. As discussed, we would like to make an audio recording of this session to make sure we have understood everything correctly. We will not share these recordings outside of the official study team, and your name will not appear in any of the files related to this session, unless you have given us permission to use it. Everything you say is strictly confidential.

Of course, if you no longer want to participate—or want to stop at any point in the session—that is completely fine, please feel free to tell me at any time.

Do you have any questions about what I’ve just said? I will begin the recording now.

**Question Guide:**

1. **Background questions**

- Please describe your current role and responsibilities.
- How long have you been in this position?

[Please continue to the relevant key-informant-specific guide below.]

1. **Educators**

- About how many students from Arab backgrounds have you taught? To your knowledge, how many of these have been refugees or asylum-seekers?
- What would you say it is like for a refugee student of Arab background to attend school in this district?
  - How do you think their experiences in the school compare to those of other students?
  - What are some of the greatest challenges that these students face compared to their classmates? [For example: language limitations, cultural differences, level of education, attendance, behavioral issues, homework completion, etc.]
  - What do you think contributes to these challenges?
  - What are some ways that you think refugee students benefit the school learning environment, if at all?
- What has your experience teaching these students been like so far?
  - How has this experience compared with teaching your other students?
  - What has been challenging about teaching these students? [repeat sequence below for each challenge mentioned.]
    - [For example: language limitations, cultural differences, level of education, attendance, motivation, behavioral or psychological issues, homework completion, parental engagement, etc.]
    - What do you think contributes to this challenge?
    - What are some ways that you have responded to this challenge?
    - How has this strategy worked?
  - What are ways that these students have benefited your classroom?
    - [For example: bringing new perspectives, experiences, or knowledge sets].
- What has been your experience interacting with the families of refugee students from Arab backgrounds?
  - What have you observed about the involvement of caregivers and other family members in their children’s education?
  - What do you think contributes to this involvement?
  - What do you think can be done to improve the involvement of caregivers and other family members in their children’s education?
- How have your students from Arab backgrounds interacted with the other students in general?
  - How receptive of refugee students from Arab backgrounds would you say the other students have been?
  - Have you observed any trends in the friendships that refugee students from Arab backgrounds are making?
    - For example, do the majority tend to make friends with students from similar backgrounds or different backgrounds?
    - What do you think contributes to whether an Arab student integrates well with other students?
    - How well are female students from Arab backgrounds integrating into the school? What are some particular challenges for girls?
    - How about male students, how well are they integrating? What are some particular challenges for boys?
  - Can you think of instances of refugee students not getting along with other students? If so, please describe an example without naming any students.
  - How concerned are you with the risk of discrimination against refugee students?
    - For instance, have you noticed any bullying of refugee students in particular or fighting between refugee students and other students?
- What has been the role of the school administration in supporting refugee students from Arab backgrounds to integrate into their new communities?
  - What are some ways that the school administration has supported refugee students and their families? [For example, more resources for ESL, hiring special interpreters and counselors, engagement with parents, etc.]
    - Which of these supports are specific to students of Arab backgrounds?
  - What are some of the ways that the school administration has supported teachers to better serve refugee students? [For example, trainings, specialized materials, etc.]
    - Which of these supports are specific to students of Arab backgrounds?
  - How would you say these strategies are working?
  - What would you change about these current strategies and why?
  - What else do you think can be done in the school to support refugee students and their families?
  - What else do you think could be done to support teachers?
  - What would be needed in order to put these strategies in place?

1. **Service providers [e.g., counselors, mental health providers, nurses, NGO employees, etc.]**

- How do your roles and responsibilities extend to supporting refugee students of Arab backgrounds?

- What has your experience been of supporting refugee youth with Arab backgrounds in this role?
  - About how many of these individuals have you worked with?
  - What are common issues that you have worked on with these individuals?
  - How has this experience compared with individuals from other backgrounds?
  - What has been challenging about serving these individuals [repeat sequence below for each challenge mentioned]?
    - [For example: language limitations, cultural differences, willingness to seek help, high psychological distress, etc.]
    - What do you think contributes to this challenge?
    - What are some ways that you have responded to this challenge?
    - How has this strategy worked?
- What has your experience been working with the families of refugee youth from Arab backgrounds?
  - In what ways have you interacted with these caregivers and other family members, if any?
  - In your experience, what is the role of caregivers in their children’s engagement with services?
  - What do you think contributes to this role of caregivers?
  - What kinds of challenges have you encountered in interacting with these caregivers and other family members?

**[If interviewing in school-based setting]**

- What would you say it is like for a refugee student of Arab background to attend school in this district?
  - How do you think their experiences in the school compare to those of other students?
  - What are some of the greatest challenges that these students face compared to their classmates? [For example: language limitations, cultural differences, level of education, attendance, behavioral issues, homework completion, etc.]
  - What do you think contributes to these challenges?
  - What are some ways that you think refugee students benefit the school learning environment, if at all?
- What has been the role of the school administration in supporting refugee students from Arab backgrounds?
  - What are some ways that the school administration has supported refugee students and their families?
    - Which of these supports are specific to students of Arab backgrounds?
  - What are some of the ways that the school administration has supported you to better serve refugee students? [For example, trainings, specialized materials, etc.].
    - Which of these supports are specific to students of Arab backgrounds?
  - How would you say these strategies are working?
  - What would you change about these current strategies and why?
  - What else do you think can be done to support refugee students and their families?
  - What else do you think could be done to support providers like yourself?
  - What would be needed in order to put these strategies in place?
- In addition to the services you provide, what are some other means of support that refugee students and their families have access to?
  - Which services would you recommend to students and/or their families?
  - Which services would you not recommend and why?

1. **Administrators [e.g., principals, superintendents, and other members of district leadership]**

- As an administrator, what are your responsibilities related to serving the refugee student population?
  - How does your work related to this portion of the student body compare to the rest of your responsibilities?
- What would you say it is like for a refugee student of Arab background to attend school in this district?
  - How do you think their experiences in the school compare to those of other students?
  - What are some of the greatest challenges that these students face compared to their classmates? [For example: language limitations, cultural differences, level of education, attendance, behavioral issues, homework completion, etc.]
  - What do you think contributes to these challenges?
  - What are some ways that you think refugee students benefit the school learning environment, if at all?
- What are some of the current efforts that your team is making at the district level to address the needs of refugee students? [repeat sequence below for each relevant initiative mentioned.]
  - Is this effort specifically focused on serving refugees?
  - How did this effort begin?
  - Who is involved in this effort and who leads it?
  - What organizations do you work with?
  - Where does support for this effort come from?
  - What are some benefits of this effort you’ve seen so far?
  - What are some challenges you’ve seen so far?
  - How are you addressing these challenges?
  - Do you have any documentation about these efforts?
- How does your office work with outside organizations to plan and/or implement such activities?
  - What does a constructive relationship with an outside organization look like?
  - What are some common challenges to working with outside organizations on these issues?
- How does your team currently work with individual high schools to address the needs of refugee students?
  - What kind of support do you provide for individual high schools? Who in those schools does your team work with?
  - How would you compare the efforts of different high schools in the district in this regard? What factors contribute to some schools being more receptive to newcomers than others?
- Aside from these current initiatives, what potential future efforts are being considered?
- What kinds of additional support would you ideally have to improve your efforts to address the needs of refugee students with Arab backgrounds?
  - Who would this support come from?
  - What would be needed to receive these means of support?
- What advice would you give to the administrator of a school that has just recently started receiving refugee students?

1. **Government officials [District/county/municipal levels]**

- What are your responsibilities related to serving the refugee population in this area?
  - How would you describe the current situation regarding refugee resettlement in this area?
  - How would you characterize the attitude of the general population in this area regarding refugee resettlement?
  - What are some common views that the general public here expresses about refugees of Arab origin?
- What are some of the activities that your office is currently undertaking related to resettled refugee youth?
  - Who is this activity specifically designed to serve? Does it have a specific focus on refugee youth?
  - When and how did this effort begin?
  - Who is involved in this effort and who leads it?
  - What agencies and/or organizations do you work with on this activity?
  - Where does support for this effort come from?
  - What are some benefits of this effort you’ve seen so far?
  - What are some challenges you’ve seen so far?
  - How are you addressing these challenges?
  - Do you have any documentation about these efforts?
- How does your office work with outside organizations to plan and/or implement such activities?
  - What does a constructive relationship with an outside organization look like?
  - What are some common challenges to working with outside organizations on these issues?
- Aside from these current initiatives, what potential future efforts are being considered?
- What kinds of additional support would you ideally have to improve your efforts to address the needs of refugee students with Arab backgrounds?
  - Who would this support come from?
  - What would be needed to receive these means of support?
- What would you say are the most important factors influencing your efforts to address the needs of refugee youth in this area?
  - For instance, how do municipal-level policies affect the efforts you can undertake? [E.g., Mayoral leadership, budget planning, etc.]
  - How do factors at the county level affect the efforts you can undertake?
  - How about the state level? And the national level?

**Closing remarks:**

That is the end of the interview. Thank you so much for your patience and cooperation. This has been an incredibly rich conversation, and I really appreciate your time and willingness to speak. Before we wrap up, do you have any questions for me?

If something comes up later, please feel free to contact me at [provide contact information]. Please remember that everything we’ve discussed today will remain strictly confidential.

Finally, after having finished this interview, if there is someone else in the area that you think we should speak with about these matters, please let us know.

Thank you again and have a great day. [Stop recording.]
